# Apocrine Breast Carcinoma With an Atypical Immunohistochemical Profile: A Case Report

**DOI:** 10.7759/cureus.89193

**Published:** 2025-08-01

**Authors:** Mohammed El Masadi, Sofia Elouaouch, Fatima Zahra Bouayed, Ahmed BenSghier, Soufiane Berhili, Mohamed Moukhlissi, Loubna Mezouar

**Affiliations:** 1 Radiation Oncology, Mohammed VI University Hospital, Faculty of Medicine and Pharmacy of Oujda, Mohammed First University of Oujda, Oujda, MAR; 2 Radiation Oncology, Centre Hospitalier Universitaire Mohammed VI, Oujda, MAR

**Keywords:** androgen receptor, apocrine breast carcinoma, atypical immunohistochemistry, breast cancer, radiation therapy

## Abstract

Breast cancer is the most common malignancy among women worldwide and encompasses a wide variety of histopathological subtypes. While invasive ductal carcinoma (IDC) represents the most prevalent form, rare variants such as apocrine carcinoma (AC) also warrant particular attention. Classically, AC of the breast is defined by apocrine morphology, negativity for hormonal receptors (estrogen receptor (ER), progesterone receptor (PR)), and strong positivity for the androgen receptor (AR). However, the present case involving a 37-year-old woman who detected a nodule at the junction of the lower quadrants of the right breast illustrates an atypical variant of this tumor subtype. Histological examination revealed two lesions displaying typical apocrine carcinomatous proliferation but with an unusual immunohistochemical profile: ER expression at 80%, PR expression at 5%, and human epidermal growth factor receptor 2 (HER2) overexpression. This deviation from the classical apocrine profile highlights the biological heterogeneity of these tumors and underscores the importance of thorough characterization to optimize therapeutic management.

## Introduction

Apocrine carcinoma (AC) of the breast is defined by the presence of apocrine differentiation in at least 90% of tumor cells [1]. It is a rare subtype of breast cancer, accounting for approximately 0.5% to 4% of invasive breast carcinomas in women [2-4].

Histologically, AC displays a tumor architecture similar to that of invasive ductal carcinoma of no special type (IDC-NST). Its distinctive features lie primarily in its cytological characteristics. The tumor cells exhibit marked apocrine differentiation, characterized by abundant granular eosinophilic cytoplasm and prominent, often multiple nucleoli [5].

Recent immunohistochemical data indicate that this tumor type is generally negative for classical hormone receptors such as estrogen receptor (ER) and progesterone receptor (PR), while frequently expressing androgen receptors (AR), corresponding to an ER−/PR−/AR+ profile. In addition, the expression of gross cystic disease fluid protein-15 (GCDFP-15), a typical marker of apocrine lesions, is commonly observed [6,7].

In this context, we report the diagnostic approach undertaken in a 37-year-old female patient presenting with a rare morphological entity: invasive breast carcinoma with apocrine differentiation.

## Case presentation

A 37-year-old female patient, with a past medical history notable for an incidentally diagnosed human immunodeficiency virus (HIV) infection during pre-therapeutic assessment and a family history of colon cancer (father), presented with an 11-month history of a palpable nodule located at the junction of the lower quadrants of the right breast. The lesion was discovered by self-examination, without associated mastalgia or nipple discharge.

**Figure 1:**
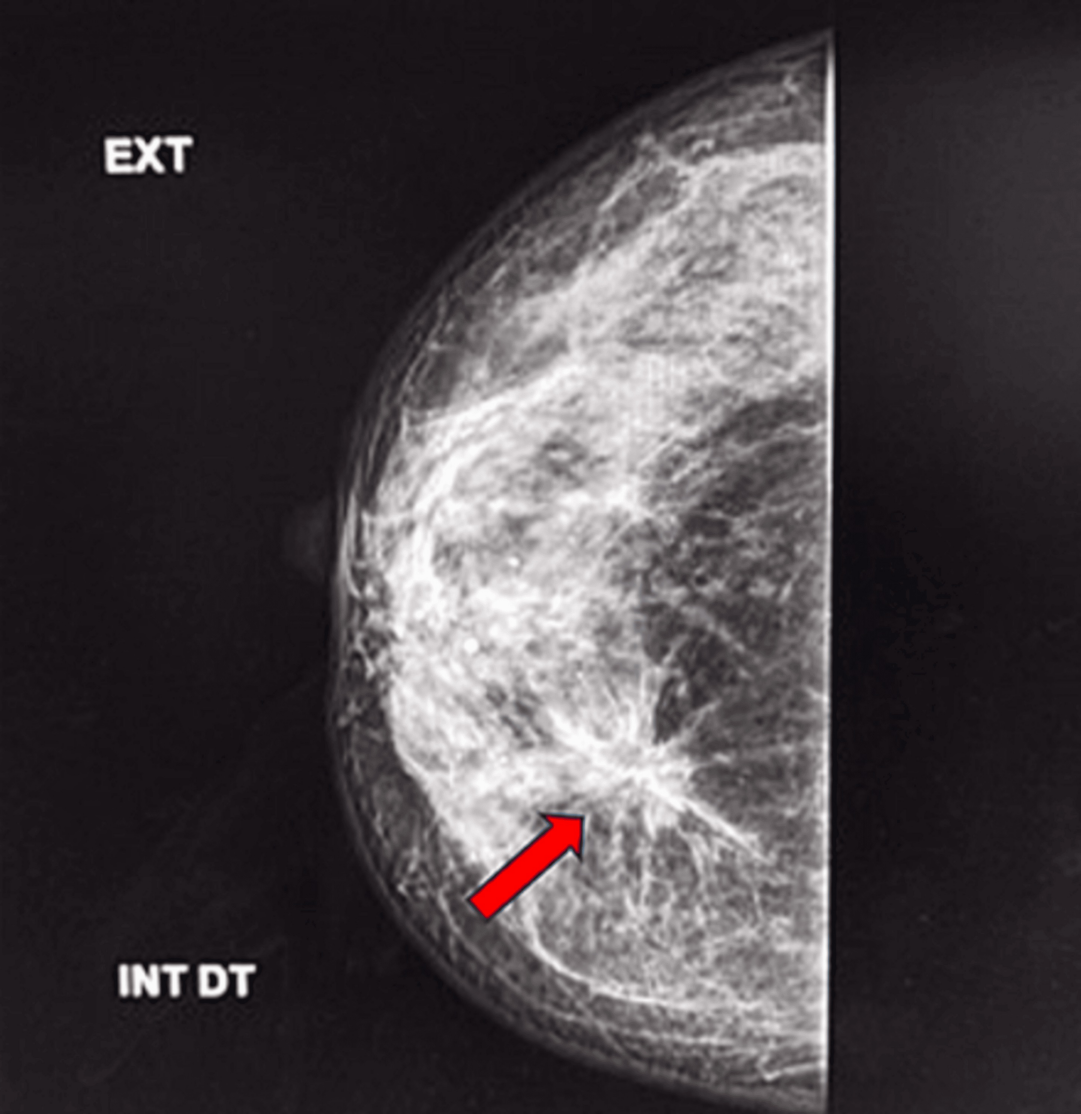
Mammographic view Right breast of type B. Two masses highly suspicious for malignancy, associated with microcalcifications, were identified and classified as BI-RADS 4C (red arrow). BI-RADS: Breast Imaging-Reporting and Data System

Physical examination revealed a 2 cm, ill-defined, mobile, firm, and non-tender nodule at the junction of the lower quadrants of the right breast, with no palpable axillary lymphadenopathy. Mammography identified two masses highly suspicious for malignancy, associated with microcalcifications, categorized as Breast Imaging Reporting and Data System (BI-RADS) 4C (Figure 1).

A core biopsy of the right breast showed a poorly differentiated carcinoma suggestive of either lobular carcinoma or IDC-NST, graded as Scarff-Bloom-Richardson (SBR) grade 2. The staging workup, including a thoraco-abdomino-pelvic computed tomography (CT) scan, was unremarkable.

The patient underwent a right radical mastectomy with axillary lymph node dissection. Macroscopically, two tumors were found: one located 3 cm medial to the nipple, measuring 3 × 1.5 × 1 cm, and a second tumor located 1 cm from the upper margin, measuring 3 × 2 × 1.5 cm. Both lesions appeared whitish, fleshy, and poorly circumscribed.

**Figure 2:**
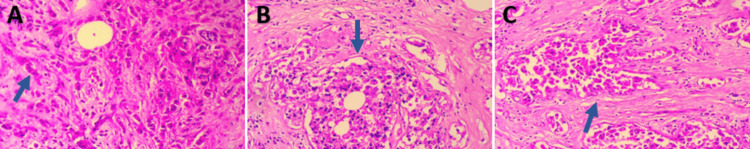
Histopathological analysis A: histological image showing an apocrine carcinoma with trabecular architecture (blue arrow); B: histological image highlighting the cytological features of apocrine carcinoma (blue arrow); C: histological image displaying an in situ apocrine carcinoma with solid architecture (blue arrow) (HE ×100).

Histopathological examination of both tumors revealed an identical morphology characterized by AC, composed of plasmacytoid and globular cells with abundant eosinophilic cytoplasm and atypical nuclei, which were generally hyperchromatic and pyknotic, occasionally vesicular with fine nucleoli. Mitoses were infrequent (<4 per 10 high-power fields). The tumor cells were predominantly arranged in thick trabeculae without tubular formation, sometimes exhibiting a lobular single-file pattern, embedded in a hyalinized and sclerotic fibrous stroma containing mononuclear inflammatory cells (Figure 2).

No evidence of vascular emboli was observed. A small solid-type in situ component was also noted. The nipple showed no signs of Paget’s disease, and the subareolar ducts were not involved. Surgical margins were negative and sufficiently clear. Among the 20 dissected lymph nodes, three harbored metastatic deposits. The tumor was staged as pT2N1cM0 according to the tumor, node, metastasis (TNM) classification system (Classification of Malignant Tumors).

**Figure 3:**
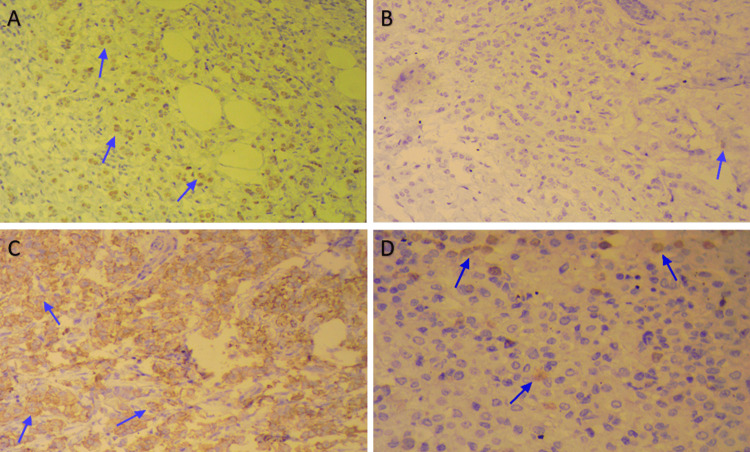
Immunohistochemistry (IHC) A: estrogen receptors (ER+) (80%), positive nuclear staining in approximately 80% of tumor cells, with weak to moderate intensity (score 1+ to 2+) (blue arrows); B: progesterone receptors (PR–) (5%), positive nuclear staining in about 5% of tumor cells, with weak intensity (score 1+) (blue arrow); C: human epidermal growth factor receptor 2 (HER2) (HercepTest®, HER2+) confirmed by fluorescence in situ hybridization (FISH), incomplete and weak to moderate membranous staining observed in more than 50% of tumor cells (blue arrows); D: proliferation Index (Ki-67 = 15%), the tumor proliferation index, assessed by Ki-67 immunostaining, was estimated at 15% (blue arrows).

Immunohistochemical profiling revealed 80% positivity for ER, 5% positivity for PR, and human epidermal growth factor receptor 2 (HER2) overexpression confirmed by both a positive HercepTest® (Agilent Technologies, CA, USA) and fluorescence in situ hybridization (FISH). The Ki-67 proliferation index was estimated at 15% (Figure 3).

AR testing was not performed. Following the surgical procedure, the patient received adjuvant chemotherapy consisting of four cycles of an anthracycline-cyclophosphamide combination, followed by three cycles of paclitaxel in combination with trastuzumab (Herceptin). The anthracycline-cyclophosphamide regimen was administered every three weeks, over three consecutive days per cycle. Twenty-one days after the final cycle, paclitaxel was initiated on a tri-weekly schedule. Trastuzumab was administered concurrently during the taxane phase and continued as maintenance monotherapy after completion of chemotherapy.

Three weeks after the final paclitaxel cycle, the patient underwent adjuvant radiotherapy targeting the right supraclavicular region and ipsilateral chest wall. The regimen consisted of a total dose of 42 Gy delivered in 15 fractions of 2.8 Gy [8].

Radiotherapy was completed successfully without clinical complications. Adjuvant endocrine therapy with an anti-estrogen agent (tamoxifen) was prescribed at a dose of 20 mg/day for five years.

The follow-up protocol included clinical examination every three months, with a mammogram scheduled six months after completion of irradiation. At seven months of follow-up, the patient remained in remission, with no evidence of locoregional recurrence or distant metastasis.

## Discussion

AC of the breast is defined by the presence of apocrine cytological and immunohistochemical features in over 90% of tumor cells [1]. Its prevalence, based on published series relying exclusively on light microscopy, is estimated to be between less than 1% and 4% of breast cancers [1-3]. Regarding age at diagnosis, no significant difference has been reported between apocrine ductal carcinomas and their non-apocrine counterparts. The reported age range spans from 19 to 86 years, with a mean age of around 52 years, consistent with the patient described in the present case [9].

Pathologically, these tumors often exhibit an architectural pattern similar to IDC-NST but are distinguished by characteristic cytological features: abundant, granular, eosinophilic cytoplasm and round to oval nuclei with prominent, occasionally multiple nucleoli [5].

Immunohistochemistry is crucial for confirming diagnoses, with ACs typically being negative for ER and PR, but strongly positive for AR, and frequently expressing the GCDFP-15 protein [6,7].

Macroscopically, AC lacks specific features; invasive tumors usually present as firm, indurated, poorly circumscribed masses with a whitish or grayish appearance [1], consistent with findings in the current case. Histologically, apocrine differentiation may be identified irrespective of histological subtype or tumor grade and is reported in various breast carcinoma subtypes, including ductal, lobular, tubular, medullary, papillary, micropapillary, and neuroendocrine carcinomas. The diagnosis of AC primarily relies on the cytological characteristics of tumor cells rather than histological origin [1].

Clinically and radiologically, AC is indistinguishable from other non-apocrine breast carcinomas, commonly presenting as a palpable nodule detected by self-examination or screening mammography [1]. Anatomical distribution is not distinct, although most tumors localize to the upper outer quadrant of the breast [1]. Bilateral ACs are rare [1]. In the present case, the carcinoma was detected by self-palpation of a nodule located at the junction of the lower quadrants of the right breast.

The management of AC of the breast remains challenging due to its rarity, representing less than 1% of invasive female breast cancers [10]. In the absence of validated, specific therapeutic protocols, clinical management primarily follows guidelines established for other invasive breast cancer subtypes, notably IDC-NST [11]. In the majority of cases of breast cancer, surgery is the standard initial approach, including either lumpectomy or mastectomy, often combined with axillary lymph node dissection depending on local extension [12].

Adjuvant treatment with chemotherapy, radiotherapy, or hormone therapy is individualized based on the tumor’s immunohistochemical profile [11]. The majority of ACs are negative for hormone receptors (ER-/PR-) [10]. However, when tumors express HER2 or AR, targeted therapies may be considered, including anti-HER2 treatment or AR-directed hormonal therapy, respectively [12].

The long-term prognosis of AC remains controversial due to its rarity and the heterogeneity of reported cohorts [13]. Nevertheless, current data suggest that overall survival (OS) and disease-specific survival (DSS) of AC are generally comparable to, or slightly better than, those observed in IDCs of no special type, particularly in AR-positive cases [14].

In this context, Kaplan-Meier survival curves were used to analyze OS and DSS according to different histological subtypes. The results showed that AC was associated with lower OS and DSS compared to IDC, with p-values of 0.006 and 0.012, respectively. However, after adjustment for clinical and pathological variables, including age, stage, and receptor status (ER, PR, and HER2), no statistically significant differences were observed for OS (p = 0.116) or DSS (p = 0.181). These findings suggest that the seemingly poorer prognosis of AC is primarily attributable to its biological characteristics at diagnosis, rather than to an intrinsically more aggressive behavior [15].

## Conclusions

AC of the breast, although rare, represents a distinct histological subtype, classically characterized by negativity for hormone receptors and strong expression of the AR. The present case illustrates an atypical form with high ER expression and HER2 overexpression, reflecting significant biological heterogeneity within this tumor type. This variability highlights the need for comprehensive immunohistochemical evaluation to ensure accurate diagnosis and tailored therapeutic management, particularly regarding targeted treatment options. A better understanding of the immunological profiles of ACs will contribute to improved classification and clinical management of these tumors.
